# Supplementary material for: PRDM1/BLIMP1 induces cancer immune evasion by modulating the USP22-SPI1-PD-L1 axis in hepatocellular carcinoma cells
Source: Nat Commun. 2022 Dec 12;13:7677. doi: 10.1038/s41467-022-35469-x (PMC9744896; doi:10.1038/s41467-022-35469-x)
Supplement: Supplementary file 1 — Supplementary Information [file 41467_2022_35469_MOESM1_ESM.pdf]

Supplementary information

PRDM1/BLIMP1 induces cancer immune evasion by modulating the USP22-SPI1-PD-L1 axis in hepatocellular carcinoma cells

Qing Li[1,†]· Liren Zhang[1,†]· Wenhua You[2,3,†]· Jiali Xu[4,†]· Jingjing Dai[5,†]· Dongxu Hua[6,†]· Ruizhi Zhang[1]· Feifan Yao[1]· Suiqing zhou[1]· Wei Huang[7]· Yongjiu Dai[1]· Yu Zhang[7]· Tasiken Baheti[7]· Xiaofeng Qian[1]· Liyong Pu[1]· Jing Xu[8,*]· Yongxiang Xia[1,*]· Chuanyong Zhang[1,*]· Jinhai Tang[9,*]· Xuehao Wang[1,*]

[1]Hepatobiliary Center, The First Affiliated Hospital of Nanjing Medical University, Key Laboratory of Liver Transplantation, Chinese Academy of Medical Sciences, NHC Key Laboratory of Living Donor Liver Transplantation (Nanjing Medical University), Nanjing, Jiangsu Province, China.

[2]School of Chemistry and Chemical Engineering, Southeast University, Nanjing, Jiangsu Province, China.

[3]Department of Immunology, Key Laboratory of Immune Microenvironment and Disease, Nanjing Medical University, Jiangsu Province, China.

[4]Department of Anesthesiology and Perioperative Medicine, The First Affiliated Hospital of Nanjing Medical University, Nanjing, Jiangsu Province, China.

[5]Department of Infectious Diseases, The First Affiliated Hospital of Nanjing Medical University, Nanjing, Jiangsu Province, China.

[6]The First School of Clinical Medicine, Nanjing Medical University, Nanjing, China.

[7]Department of General Surgery, The Friendship Hospital of Ili Kazakh Autonomous Prefecture; Ili & Jiangsu Joint Institute of Health, Ili, China.

[8]Department of Oncology, The First Affiliated Hospital of Nanjing Medical University, Nanjing, Jiangsu Province, China.

[9]Department of General Surgery, The First Affiliated Hospital of Nanjing Medical University, Nanjing, Jiangsu Province, China.

[†]These authors contributed equally to this work.

[*]**Correspondence to:** Xuehao Wang, **email:** wangxh@njmu.edu.cn

Jinhai Tang, **email:** jhtang@njmu.edu.cn

Chuanyong Zhang, **email:** zcy13951673178@163.com

Yongxiang Xia, **email:** yx_xia@njmu.edu.cn
Jing Xu, **email:** xujing7901@jsph.org.cn

Supplementary Figure 1

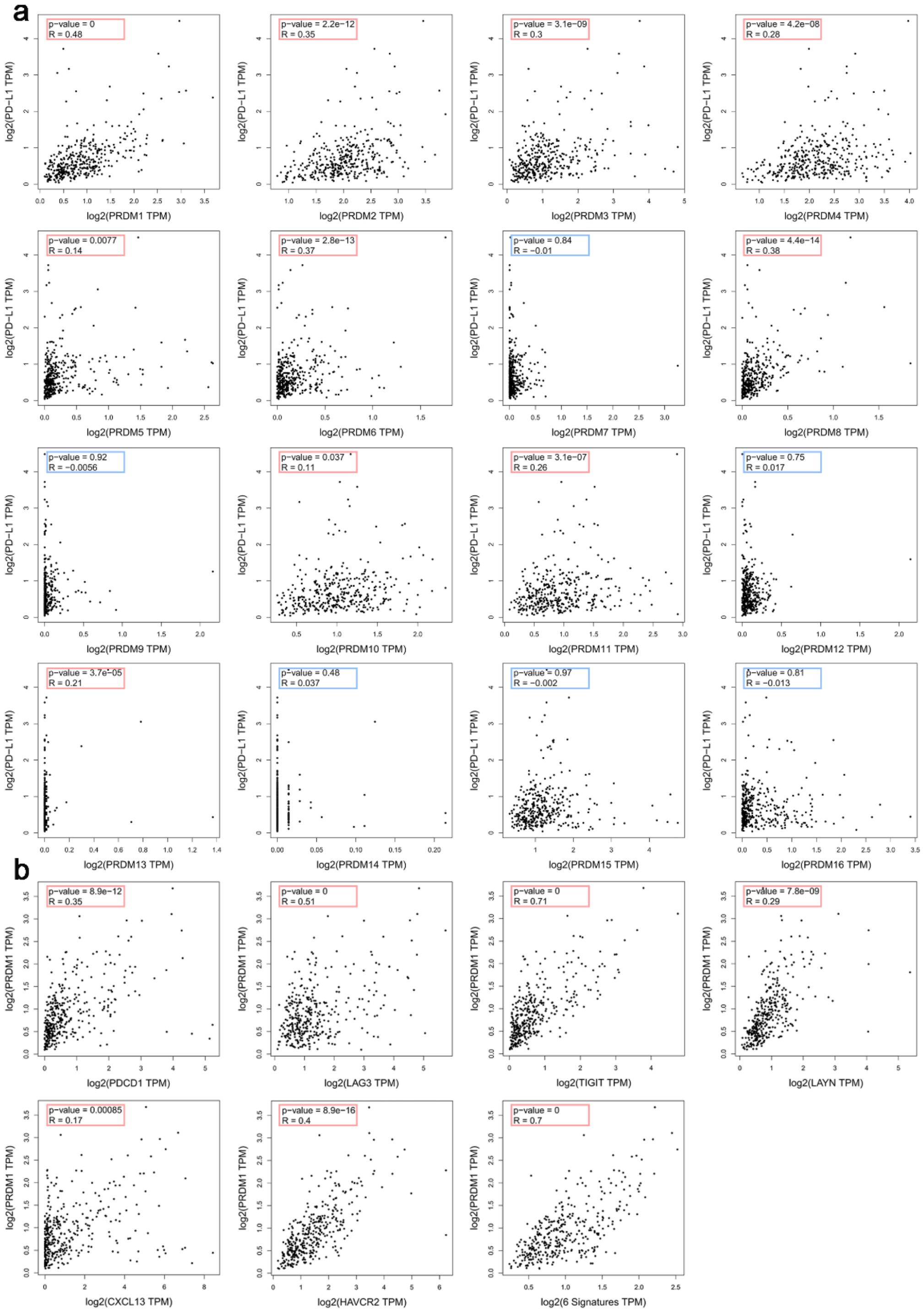

Supplementary Figure 1 *PRDM1* is a regulator of *PD-L1* expression in the GEPIA database. a The GEPIA database revealed the association of the *PRDM* family with *PD-L1*. **b** The GEPIA database revealed the association of *PRDM1* with *PDCD1*, *LAG3*, *TIGHT*, *LAYN*, *CXCL13*, *HAVCR2*, and above 6 T cell exhaustion markers. n = 369 patients. Statistical tests were two-sided. P value was determined by Pearson correlation analysis.

Supplementary Figure 2

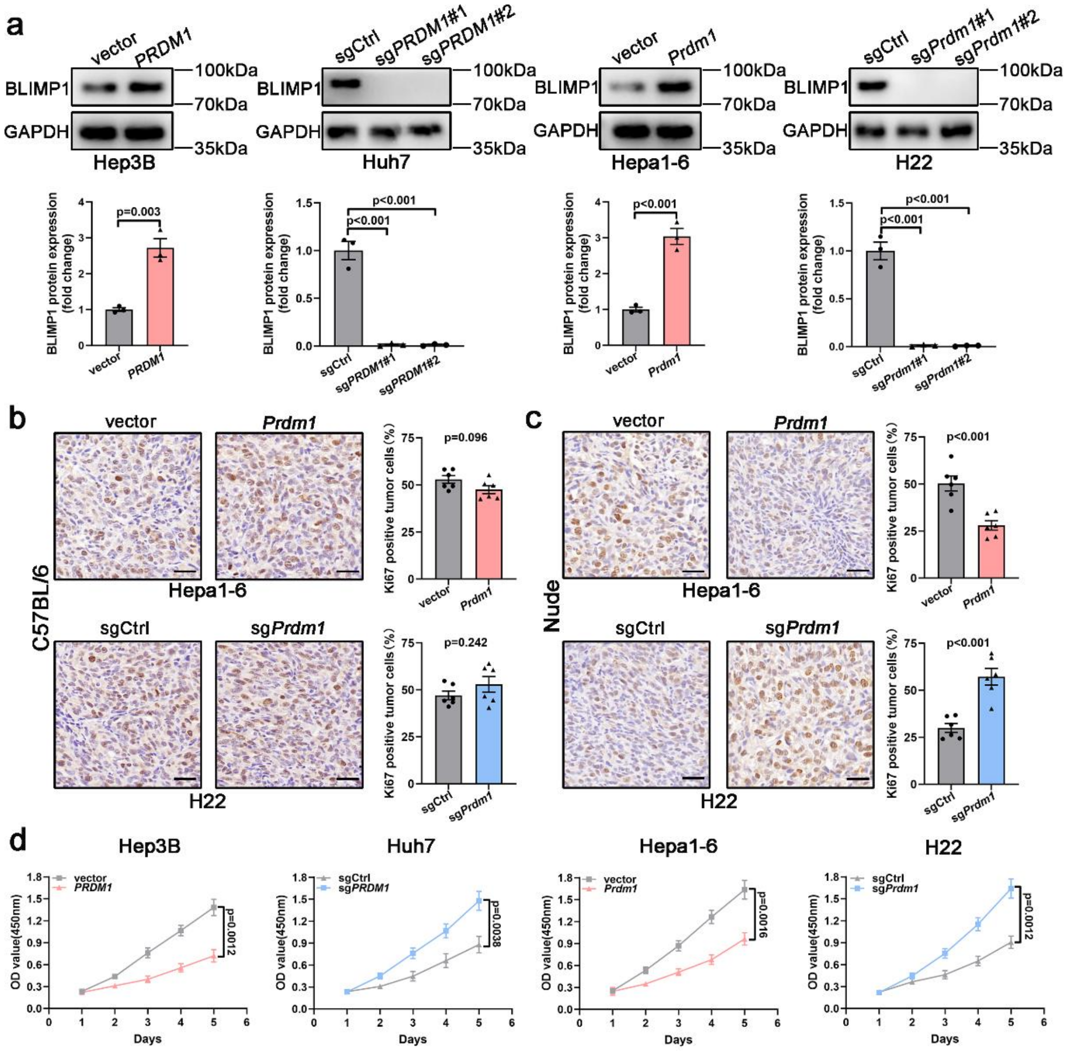

Supplementary Figure 2 ***PRDM1*** **overexpression inhibits cell-intrinsic cell growth.** **a** Western blot validation of BLIMP1 expression in the indicated HCC cells. Representative of n = 3 independent biological replicates. **b** Immunohistochemical analysis of Ki-67 expression in subcutaneous tumors collected from Hepa1-6/H22-bearing C57BL/6 mice (left) (n = 6 mice per group). Scale bar: 100 μm. The quantitative ratio of Ki-67 positive tumor cells is shown on the right. Data presented as mean ± SEM. **c** Immunohistochemical analysis of Ki-67 expression in subcutaneous tumors collected from Hepa1-6/H22-bearing BALB/c nude mice (left) (n = 6 mice per group). Scale bar: 100 μm. The quantitative ratio of Ki-67 positive tumor cells is shown on the right. Data presented as mean ± SEM. **d** CCK8 assays in the indicated HCC cells. Data presented as mean ± SEM (n = 3 independent biological replicates). P value was determined by unpaired two-sided Student’s t test with no correction for multiple comparison. The source data are provided as a Source data file.

Supplementary Figure 3

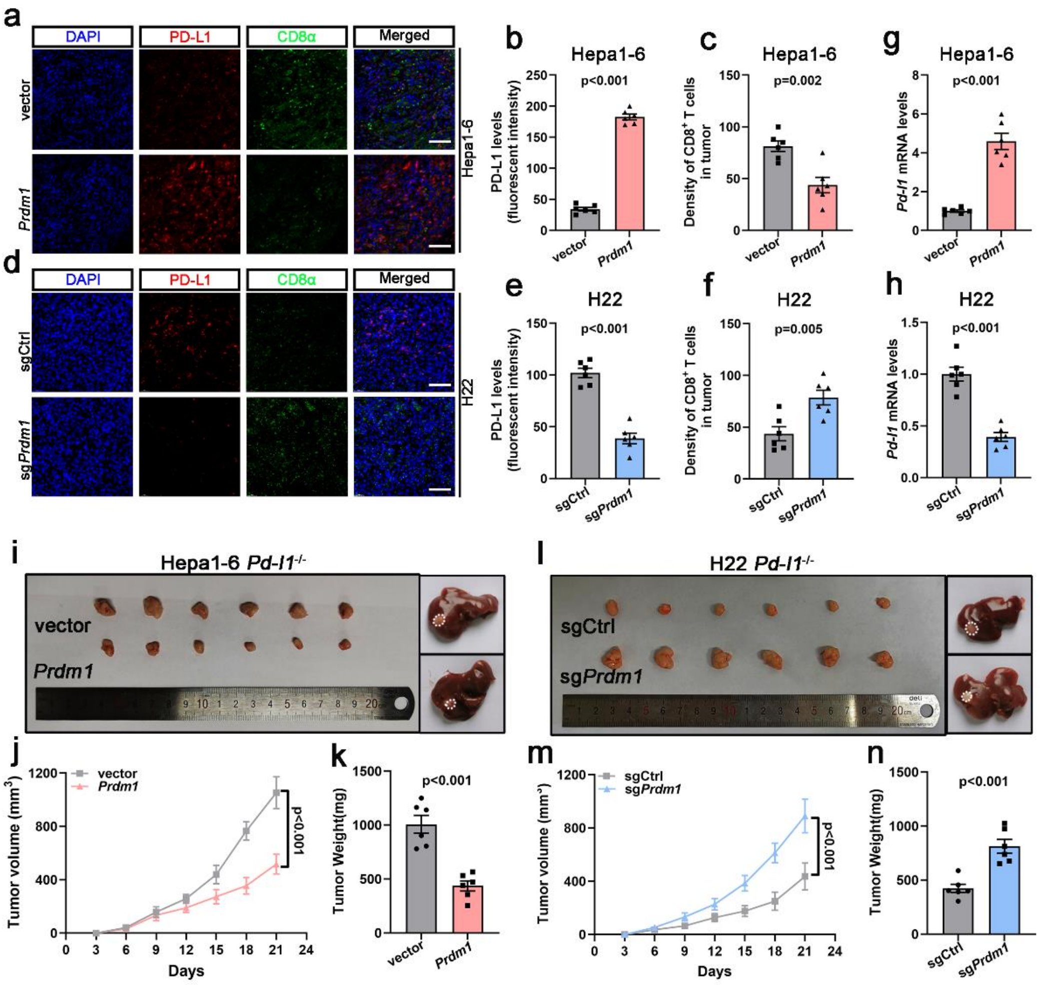

Supplementary Figure 3 ***Prdm1*****-mediated tumor immune escape is dependent on PD-L1. a-f** IF staining of PD-L1 and CD8α in subcutaneous tumors of the indicated groups and quantification of fluorescence intensity. Scale bars, 100 μm. Data presented as mean ± SEM. n = 6 mice per group. **g-h** *Pd-l1* mRNA levels in the subcutaneous tumors of indicated groups. Data presented as mean ± SEM. n = 6 mice per group. **i** Representative subcutaneous tumors (left)/orthotopic transplantation tumors (right) collected from Hepa1-6-bearing C57BL/6 mice. **j** Tumor proliferation curves of subcutaneous xenografts in Hepa1-6-bearing C57BL/6 mice. Data presented as mean ± SEM. n = 6 mice per group. **k** Tumor weight of subcutaneous tumors in Hepa1-6-bearing C57BL/6 mice. Data presented as mean ± SEM. n = 6 mice per group. **l** Representative subcutaneous tumors (left)/orthotopic transplantation tumors (right) collected from H22-bearing C57BL/6 mice. **m** Tumor proliferation curves of subcutaneous xenografts in H22-bearing C57BL/6 mice. Data presented as mean ± SEM. n = 6 mice per group. **n** Tumor weight of subcutaneous tumors in H22-bearing C57BL/6 mice. Data presented as mean ± SEM. n = 6 mice per group. P value was

determined by unpaired two-sided Student's t test. The source data are provided as a Source data file.

Supplementary Figure 4

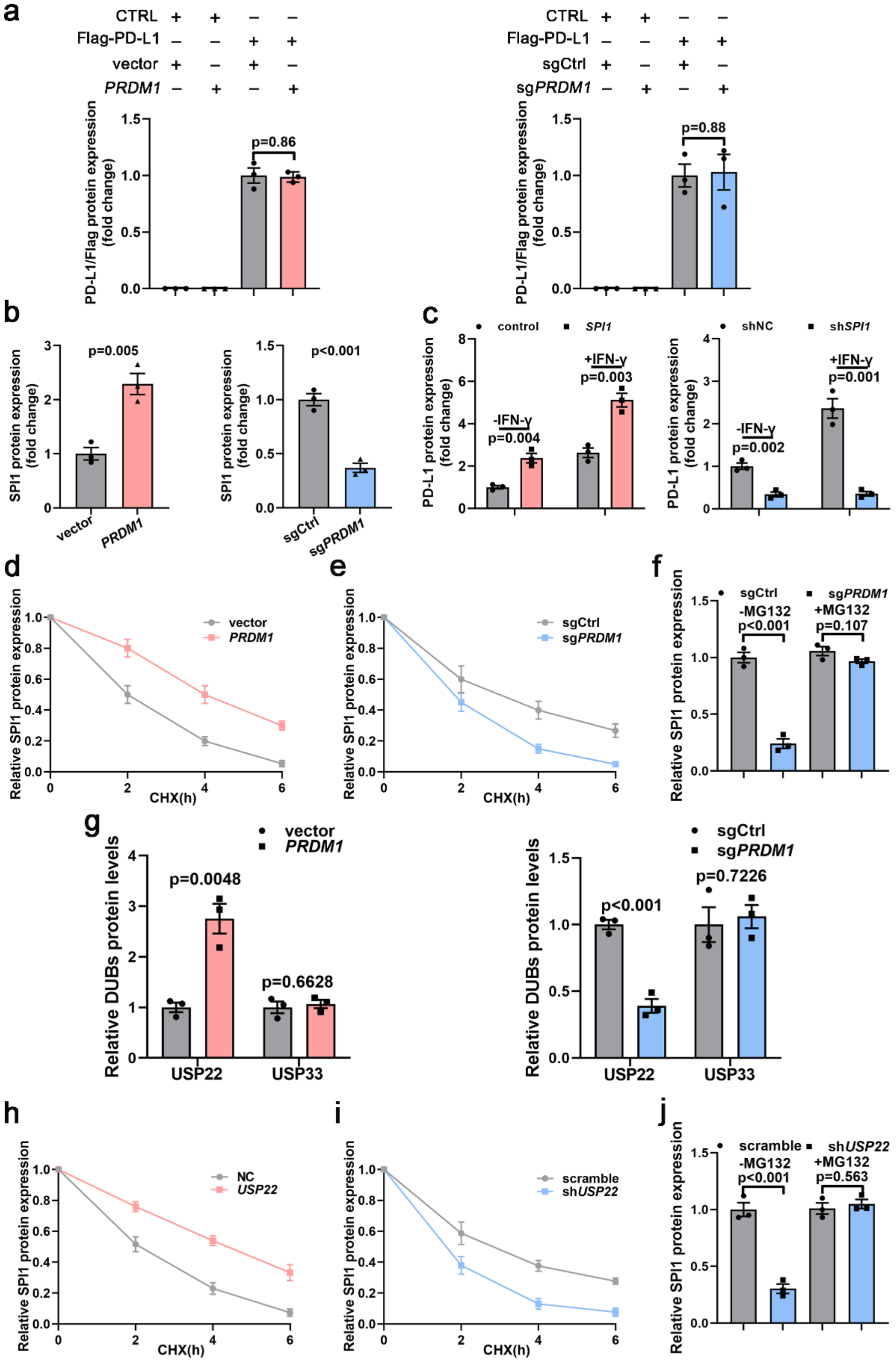

Supplementary Figure 4 Quantitative analyses of the western blot results, related to Figure 3 and Figure 4. a Quantitative analyses of the western blot results in Fig. 3a. Representative of n = 3 independent biological replicates. **b** Quantitative analyses of the western blot results in Fig. 3d. Representative of n = 3 independent biological

replicates. **c** Quantitative analyses of the western blot results in Fig. 3e. Representative of n = 3 independent biological replicates. **d-e** Quantitative analyses of the western blot results in Fig. 4a-b, respectively. Representative of n = 3 independent biological replicates. **f** Quantitative analyses of the western blot results in Fig. 4c. Representative of n = 3 independent biological replicates. **g** Quantitative analyses of the western blot results in Fig. 4g. Representative of n = 3 independent biological replicates. **h-i** Quantitative analyses of the western blot results in Fig. 4i-j, respectively. Representative of n = 3 independent biological replicates. **j** Quantitative analyses of the western blot results in Fig. 4k. Representative of n = 3 independent biological replicates. P value was determined by unpaired two-sided Student’s t test. The source data are provided as a Source data file.

Supplementary Figure 5

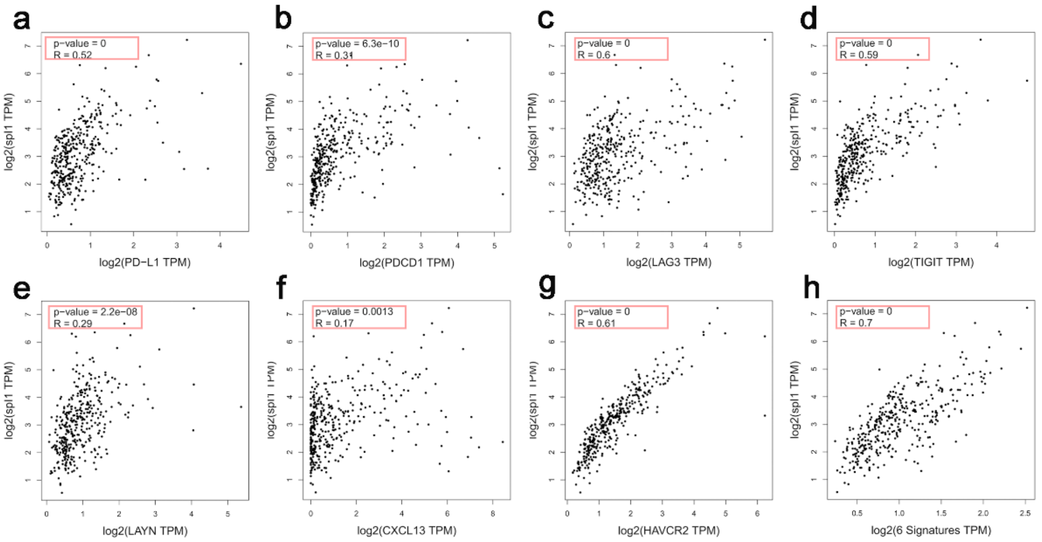

Supplementary Figure 5 *SPI1* is a regulator of *PD-L1* expression in the GEPIA database. a-g The GEPIA database revealed the association of *SPI1* with *PD-L1* (**a**), *PDCD1* (**b**), *LAG3* (**c**), *TIGHT* (**d**), *LAYN* (**e**), *CXCL13* (**f**), and *HAVCR2* (**g**). **h** The GEPIA database revealed the association of *SPI1* with the above 6 T cell exhaustion markers (*PDCD1*, *LAG3*, *TIGHT*, *LAYN*, *CXCL13*, and *HAVCR2*). n = 369 patients. Statistical tests were two-sided. P value was determined by Pearson correlation analysis.

Supplementary Figure 6

a

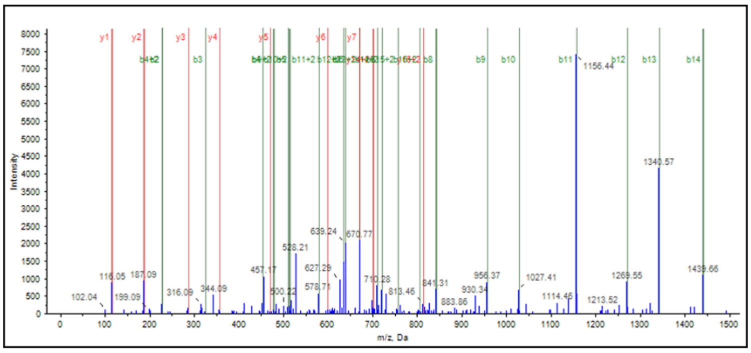

b

Fragmentation Evidence

PEPEGEAMDAELAVAP

Residue	b	b+2	y	y+2
P	98.0600	49.5337	1625.7363	813.3718
E	227.1026	114.0550	1528.6836	764.8454
P	324.1554	162.5813	1399.6410	700.3241
E	453.1980	227.1026	1302.5882	651.7977
G	510.2195	255.6134	1173.5456	587.2764
E	639.2620	320.1347	1116.5242	558.7657
A	710.2992	355.6532	987.4816	494.2444
M	841.3396	421.1735	916.4444	458.7259
D	956.3666	478.6869	785.4040	393.2056
A	1027.4037	514.2055	670.3770	335.6921
E	1156.4463	578.7268	599.3399	300.1736
L	1269.5304	635.2688	470.2973	235.6523
A	1340.5675	670.7874	357.2132	179.1103
V	1439.6359	720.3216	286.1761	143.5917
A	1510.6730	755.8401	187.1077	94.0575
P	1607.7258	804.3665	116.0706	58.5389

Supplementary Figure 6 USP22 interacts with SPI1. a Immunoprecipitation/mass spectrum (IP/MS) analysis was utilized to validate USP22 as the interacting protein of SPI1. **b** Fragmentation evidence for USP22.

Supplementary Figure 7

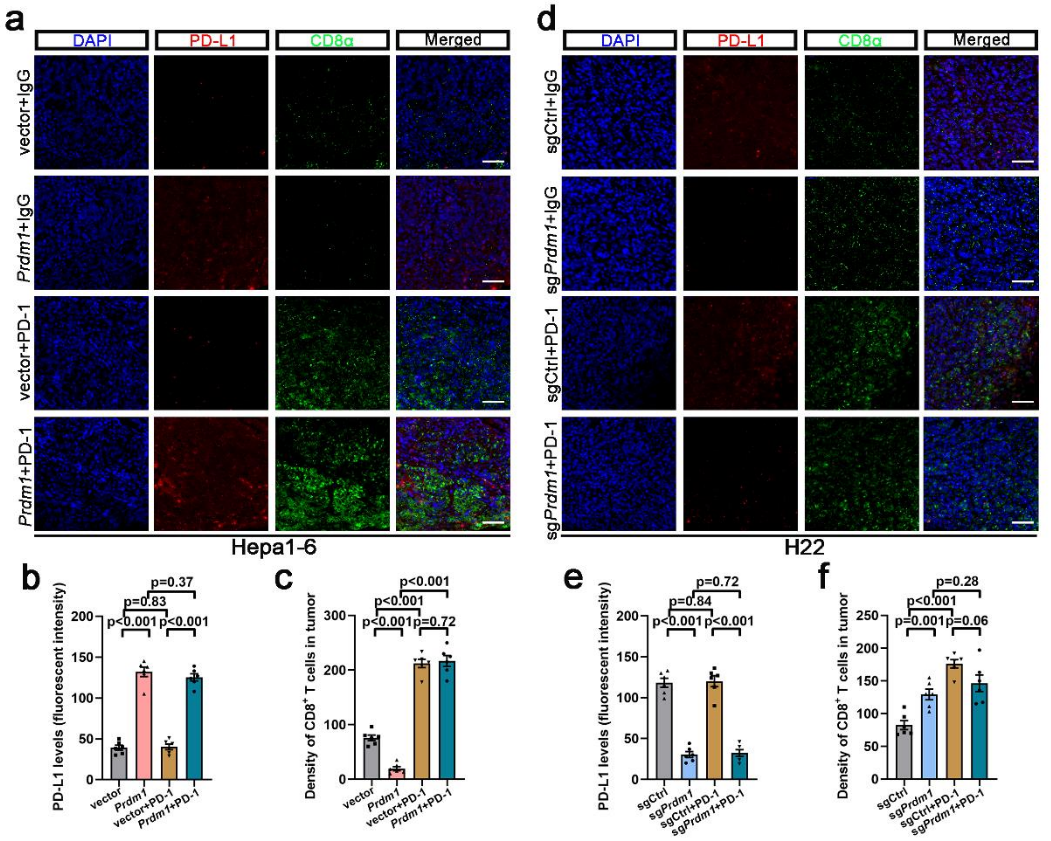

Supplementary Figure 7 Synergistic effect of *Prdm1* overexpression and PD-1 mAb dramatically augmented the infiltrated CD8⁺ T cells. a-c Immunofluorescence staining of PD-L1 and CD8α (**a**) and quantification of fluorescence intensity (**b** and **c**) in Hepa1-6 xenografts. Scale bars, 100 μm. Data presented as mean ± SEM. n = 6 mice per group. **d-f** Immunofluorescence staining of PD-L1 and CD8α (**d**) and quantification of the fluorescence intensity (**e** and **f**) in H22 xenografts. Scale bars, 100 μm. Data presented as mean ± SEM. n = 6 mice per group. P value was determined by unpaired two-sided Student's t test with no correction for multiple comparison. The source data are provided as a Source data file.

Supplementary Figure 8

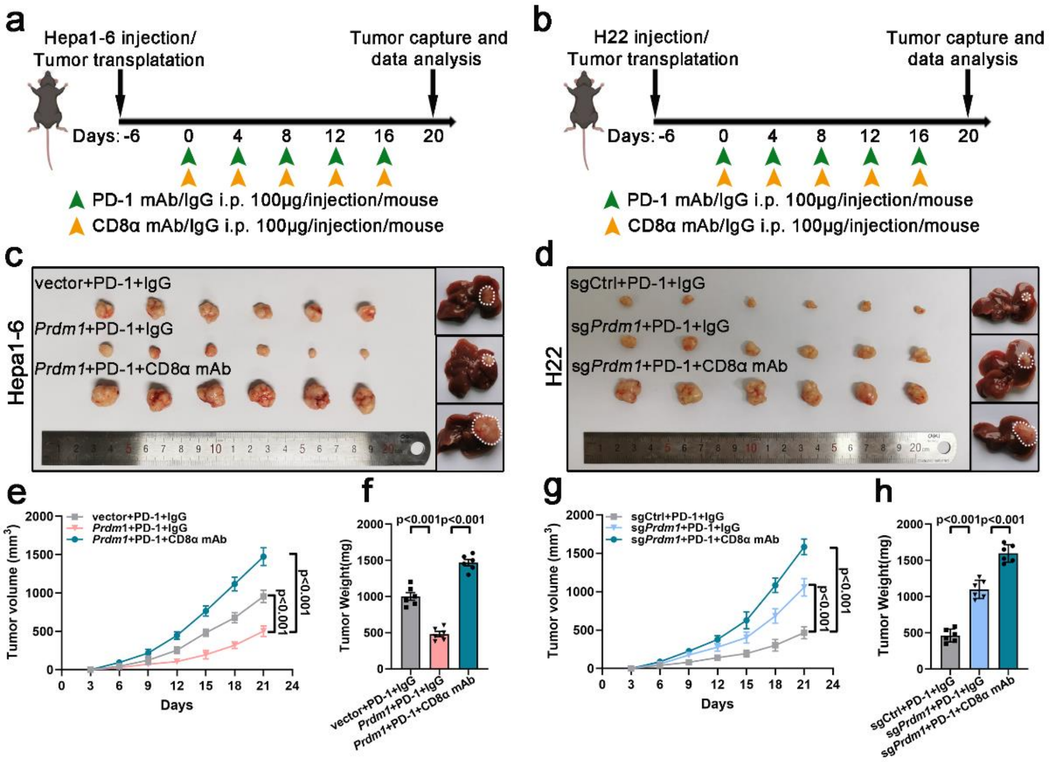

Supplementary Figure 8 CD8^{+} T cells are essential for the synergistic effect of cotreatment with *Prdm1* overexpression and PD-1 mAb. a, b Schematic view of the treatment plan in subcutaneous and orthotopic tumors. **c, d** Representative xenograft tumors (left) or orthotopic tumors (right) obtained after euthanizing the mice. **e, f** Tumor growth curves (**e**) and tumor weights (**f**) of Hepa1-6-bearing C57BL/6 mice. Data presented as mean ± SEM. n = 6 mice per group. **g-h** Tumor growth curves (**g**) and tumor weights (**h**) of H22-bearing C57BL/6 mice. Data presented as mean ± SEM. n = 6 mice per group. P value was determined by unpaired two-sided Student's t test with no correction for multiple comparison. Schematic diagrams (**a** and **b**) were created with BioRender.com. The source data are provided as a Source data file.

Supplementary Figure 9

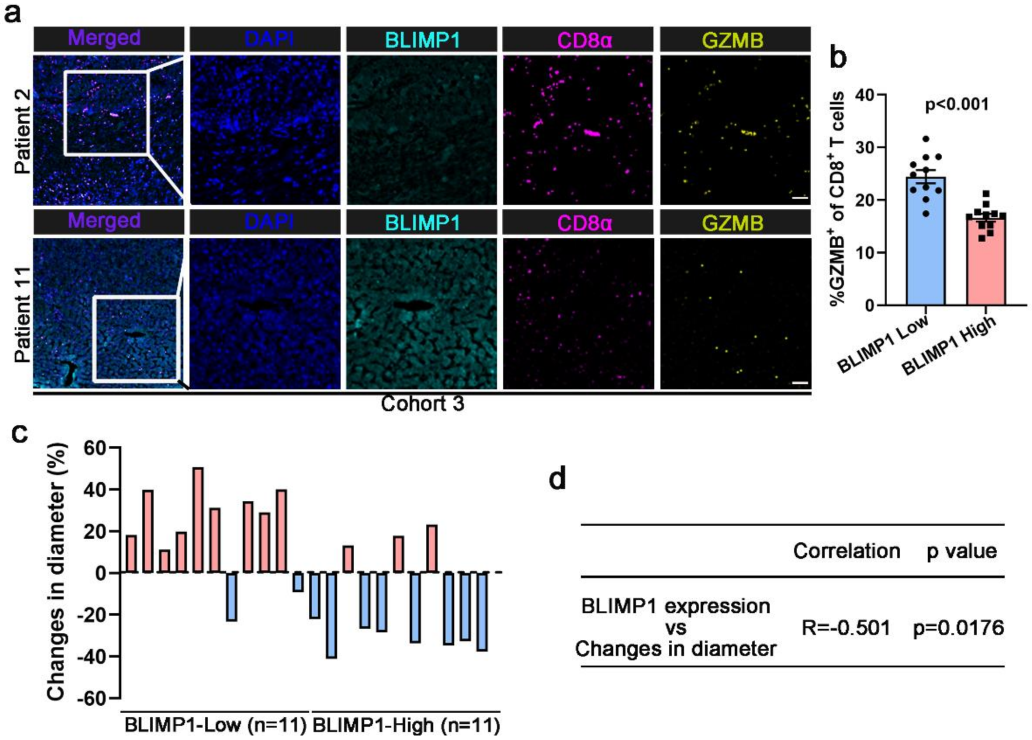

Supplementary Figure 9 *PRDM1*/BLIMP1 overexpression potentiates T-cell exhaustion. a Multi-color immunohistochemistry using HCC biopsies before PD-1 mAb-based therapies. Scale bars, 100 μm. **b** The quantitative ratio of GZMB^{+}CD8^{+} T cells. Data presented as mean ± SEM. n = 22 patients. **c** The changes in tumor diameter for patients, where those with increased tumor diameter are colored by red and those with decreased tumor diameter are colored by blue. n = 22 patients. **d** Quantitative correlation between the changes in tumor diameter and BLIMP1 expression levels. P value was determined by unpaired two-sided Student's t test (**b**) and Pearson correlation analysis (**d**). The source data are provided as a Source data file.

Supplementary Figure 10

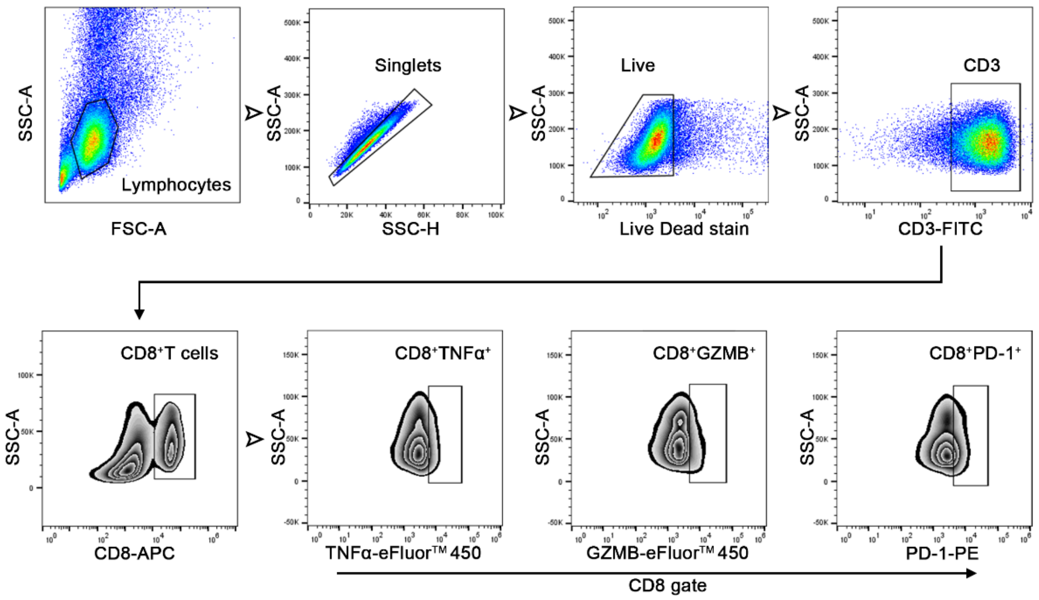

Supplementary Figure 10 Gating strategies used for flow cytometry staining. Gating strategy used to sort CD8^{+} T cells, CD8^{+}TNFα^{+} cells, CD8^{+}GZMB^{+} cells, and CD8^{+} PD1^{+} cells and for flow cytometry staining (Figure 1f, g, and k; Figure 2g and h; Figure 6i and j).

Supplementary Table 1: DUB Family

Entrez Gene ID	Current Symbol	Entrez Gene ID	Current Symbol	Entrez Gene ID	Current Symbol
1540	CYLD	10869	USP19	59343	SENP2
4287	ATXN3	10980	COPS6	64854	USP46
5713	PSMD7	10987	COPS5	78990	OTUB2
7128	TNFAIP3	11274	USP18	79101	JOSD3
7345	UCHL1	23032	USP33	79184	BRCC3
7347	UCHL3	23192	ATG4B	80124	VCPIP1
7375	USP4	23326	USP22	83844	USP26
7398	USP1	23358	USP24	84101	USP44
7874	USP7	25862	USP49	84132	USP42
8078	USP5	26054	SENP6	84196	USP48
8237	USP11	26168	SENP3	84640	USP38
8239	USP9X	27005	USP21	84669	USP32
8287	USP9Y	29761	USP25	84749	USP30
8314	BAP1	29843	SENP1	84954	MPND
8665	EIF3S5	51377	UCHL5	85015	USP45
8667	EIF3S3	51633	OTUD6B	92552	ATXN3L
8975	USP13	54726	OTUD4	114803	MYSM1
9097	USP14	54764	ZRANB1	115201	ATG4A
9098	USP6	55031	USP47	124739	USP43
9099	USP2	55230	USP40	126119	JOSD2
9100	USP10	55432	YOD1	139562	OTUD6A
9101	USP8	55593	OTUD5	158880	USP51
9736	USP34	55611	OTUB1	159195	USP54
9924	USP52	56957	OTUD7B	161725	OTUD7A
9929	JOSD1	57097	PARP11	205564	SENP5
9958	USP15	57337	SENP7	219333	USP12
9960	USP3	57478	USP31	220213	OTUD1
10208	USPL1	57558	USP35	220594	LOC220594
10213	PSMD14	57559	STAMBPL1	373509	USP50
10600	USP16	57602	USP36	373856	USP41
10617	STAMBP	57646	USP28	377630	DUB3
10713	USP39	57663	USP29	645836	LOC645836
10868	USP20	57695	USP37		

Supplementary Table 2: General Description of Patients with HCC from Nanjing Medical University

Characteristics	Cohort1	Cohort2	Cohort3
**No. of patients**	40	90	22
**Age**			
≤60	19 (47.5%)	34 (37.8%)	18 (81.8%)
>60	21 (52.5%)	56 (62.2%)	4 (18.2%)
**Gender**			
Male	30 (75.0%)	75 (83.3%)	16 (72.7%)
Female	10 (25.0%)	15 (16.7%)	6 (27.3%)
**Cirrhosis status**			
Yes	34 (85.0%)	79 (87.8%)	18 (81.8%)
No	6 (15.0%)	11 (12.2%)	4 (18.2%)
**HBs Antigen**			
Yes	36 (90.0%)	78 (86.7%)	19 (86.4%)
No	4 (10.0%)	12 (13.3%)	3 (13.6%)
**AFP**			
Negative	10 (25.0%)	19 (21.1%)	5 (22.7%)
Positive	30 (75.0%)	71 (78.9%)	17 (77.3%)
**Tumor Stage**			
I	17 (42.5%)	35 (38.9%)	0 (0%)
II	8 (20.0%)	26 (28.9%)	6 (27.3%)
III	11 (27.5%)	21 (23.3%)	14 (63.6%)
IV	4 (10.0%)	8 (8.9%)	2 (9.1%)

Supplementary Table 3: Sequences of oligonucleotides for sgRNA/shRNA

Gene	Species	Sequence (5'-3')
sg*PRDM1*	Human	sg*PRDM1*#1: GAAGTGGTGAAGCTCCCCTC<br>sg*PRDM1*#2: GCAGCCAGGTTTTGCTCCCG
sg*Prdm1*	mouse	sg*Prdm1*#1: ACACGCTTTGGACCCCTCAT<br>sg*Prdm1*#2: GAAGTGGTGGAACTCCTCTC
sh*USP22*	Human	sh*USP22*: TCGAAGAGTGGTAAGGTTCACA
sh*SPI1*	Human	sh*SPI1*: AGTCCCAGTAATGGTCGCTAT

Supplementary Table 4: Primers sequences used for real-time PCR

Gene	Species	Sequence (5'-3')
PD-L1	Human	Forward: TGGCATTTGCTGAACGCATTT
		Reverse: TGCAGCCAGGTCTAATTGTTTT
USP22	Human	Forward: GTGGACAACTGGAAGCAGAAC
		Reverse: AGGAATGCAGCCTGTTGAGG
USP33	Human	Forward: TCGGCAAGCCCTCCTAAATC
		Reverse: TTGCCAGGAATTGGCAAGGA
SPI1	Human	Forward: ATGGAAGGGTTTCCCCTCGT
		Reverse: CTGGAGCTCCGTGAAGTTGT

Supplementary Table 5: Primers sequences used for ChIP

Gene	Species	Sequence (5'-3')
SPI1/PD-L1 binding site 1	Human	Forward: AACTTGTTGTACATGTGTGTGTCA<br>Reverse: CCCGAAGTTGGGTGACTTCC
SPI1/PD-L1 binding site 2	Human	Forward: ACTTCGGGAACTTTGGGAAGTC<br>Reverse: AGGCTGACACTGCCTTGATTT
SPI1/PD-L1 binding site 3	Human	Forward: AGCCTTAATCCTTAGGGTGGC<br>Reverse: AAGAACTTCCCATCCCGAGC
SPI1/PD-L1 binding site 4	Human	Forward: GAGGAAGTCACAGAATCCACGA<br>Reverse: AAAGTCAGCAGCAGACCCAT
SPI1/PD-L1 negative control#1	Human	Forward: TTGGGGCCAAAGAGAACTCC<br>Reverse: TTATCTGGGAAATTATTGAGGCTGA
SPI1/PD-L1 negative control#2	Human	Forward: CTAGAAGTTCAGCGCGGGAT<br>Reverse: TGAATGGGCCCAAGATGACA
BLIMP1/USP22 binding site	Human	Forward: TGAGCGAGCATCAGACATGG<br>Reverse: GACAAATTGCCGAGCACAGG
BLIMP1/USP22 negative control#1	Human	Forward: AGGGCTTCACCGGGTAATTC<br>Reverse: TACTACAGCCGCAAGTTCCC
BLIMP1/USP22 negative control#2	Human	Forward: ATGTGAAGTGTCAGGCGTGT<br>Reverse: CTGATAGTGAGGGCCAACCC
