# Supplementary material for: PRDM1/BLIMP1 induces cancer immune evasion by modulating the USP22-SPI1-PD-L1 axis in hepatocellular carcinoma cells
Source: Nat Commun. 2022 Dec 12;13:7677. doi: 10.1038/s41467-022-35469-x (PMC9744896; doi:10.1038/s41467-022-35469-x)
Supplement: Supplementary file 3 — Reporting Summary [file 41467_2022_35469_MOESM3_ESM.pdf]

nature portfolio

Corresponding author(s): Xuehao Wang, Jinhai Tang, Chuanyong Zhang, Yongxiang Xia, Jing Xu

Last updated by author(s): Nov 6, 2022

Reporting Summary

Nature Portfolio wishes to improve the reproducibility of the work that we publish. This form provides structure for consistency and transparency in reporting. For further information on Nature Portfolio policies, see our Editorial Policies and the Editorial Policy Checklist.

Statistics

For all statistical analyses, confirm that the following items are present in the figure legend, table legend, main text, or Methods section.

n/a	Confirmed	
☐	☒	The exact sample size (*n*) for each experimental group/condition, given as a discrete number and unit of measurement
☐	☒	A statement on whether measurements were taken from distinct samples or whether the same sample was measured repeatedly
☐	☒	The statistical test(s) used AND whether they are one- or two-sided<br>*Only common tests should be described solely by name; describe more complex techniques in the Methods section.*
☒	☐	A description of all covariates tested
☐	☒	A description of any assumptions or corrections, such as tests of normality and adjustment for multiple comparisons
☐	☒	A full description of the statistical parameters including central tendency (e.g. means) or other basic estimates (e.g. regression coefficient) AND variation (e.g. standard deviation) or associated estimates of uncertainty (e.g. confidence intervals)
☐	☒	For null hypothesis testing, the test statistic (e.g. *F*, *t*, *r*) with confidence intervals, effect sizes, degrees of freedom and *P* value noted<br>*Give P values as exact values whenever suitable.*
☒	☐	For Bayesian analysis, information on the choice of priors and Markov chain Monte Carlo settings
☒	☐	For hierarchical and complex designs, identification of the appropriate level for tests and full reporting of outcomes
☐	☒	Estimates of effect sizes (e.g. Cohen's *d*, Pearson's *r*), indicating how they were calculated

Our web collection on statistics for biologists contains articles on many of the points above.

Software and code

Policy information about availability of computer code

Data collection	Image Lab 5.2.1; Nanodrop 2000; Leica LAS AF Lite 2.6.0.
Data analysis	ImageJ 1.8.0; Graphpad Prism 8.0; FlowJo V10.7; Proteome Discoverer 1.4; Q-Exactive mass spectrometer

For manuscripts utilizing custom algorithms or software that are central to the research but not yet described in published literature, software must be made available to editors and reviewers. We strongly encourage code deposition in a community repository (e.g. GitHub). See the Nature Portfolio guidelines for submitting code & software for further information.

Data

Policy information about availability of data

All manuscripts must include a data availability statement. This statement should provide the following information, where applicable:

- Accession codes, unique identifiers, or web links for publicly available datasets
- A description of any restrictions on data availability
- For clinical datasets or third party data, please ensure that the statement adheres to our policy

The authors declare that all data supporting the findings of this study are available within this article, its supplementary information files, and source data. The source data underlying Figs. 1-8, Supplementary Figures 1-10 are provided as a Source Data file. The mass spectrometry proteomics data in this study have been deposited to the ProteomeXchange Consortium via the PRIDE partner repository ubder accession code PXD037621 (http://www.ebi.ac.uk/pride). The remaining data are available within the Article, Supplementary Information or Source Data file. Source data are provided with this paper.

Human research participants

Policy information about studies involving human research participants and Sex and Gender in Research.

Reporting on sex and gender	Sex and gender were not considered in our study design.
Population characteristics	Three independent cohorts of HCC patients were collected from at the Hepatobiliary Center of The First Affiliated Hospital of Nanjing Medical University. Population characteristics was provided in Supplementary Table 2.
Recruitment	The use of clinical samples was approved by the Ethics Committee of The Affiliated Hospital of Nanjing Medical University. Written informed patient consent was obtained in accordance with regional regulation. The data of their clinicopathological features were anonymized. All tumor samples were confirmed by pathologists following histopathological review of H&E slides.
Ethics oversight	The use of clinical samples was approved by the Ethics Committee of The First Affiliated Hospital of Nanjing Medical University. Written informed patient consent was obtained in accordance with regional regulation.

Note that full information on the approval of the study protocol must also be provided in the manuscript.

Field-specific reporting

Please select the one below that is the best fit for your research. If you are not sure, read the appropriate sections before making your selection.

☒ Life sciences ☐ Behavioural & social sciences ☐ Ecological, evolutionary & environmental sciences

For a reference copy of the document with all sections, see nature.com/documents/nr-reporting-summary-flat.pdf

Life sciences study design

All studies must disclose on these points even when the disclosure is negative.

Sample size	No sample size calculation was performed. Sample size was determined based on the level and consistency between two different groups. All data sets include at least three biological replicates
Data exclusions	No data were excluded from analysis.
Replication	All experimental data are given including replicates. Details of experimental replicates are given in the figure legends. All reported attempts at replication were successful.
Randomization	All allocations were random in this study.
Blinding	All data collection and analysis was blinded.

Reporting for specific materials, systems and methods

We require information from authors about some types of materials, experimental systems and methods used in many studies. Here, indicate whether each material, system or method listed is relevant to your study. If you are not sure if a list item applies to your research, read the appropriate section before selecting a response.

Materials & experimental systems

n/a	Involved in the study	
☐	☒	Antibodies
☐	☒	Eukaryotic cell lines
☒	☐	Palaeontology and archaeology
☐	☒	Animals and other organisms
☒	☐	Clinical data
☒	☐	Dual use research of concern

Methods

n/a	Involved in the study	
☒	☐	ChIP-seq
☐	☒	Flow cytometry
☒	☐	MRI-based neuroimaging

Antibodies

Antibodies used

BLIMP1, ab198287, monoclonal, Abcam, Cambridge, MA, USA, 1/500 for IHC-P
BLIMP1, ab243146, monoclonal, Abcam, Cambridge, MA, USA, 1/1000 for WB
BLIMP1, ab13700, polyclonal, Abcam, Cambridge, MA, USA, 5 μg/25 μg chromatin for CHIP
USP22, ab195289, monoclonal, Abcam, Cambridge, MA, USA, 1/1000 for IHC-P, 1/2000 for WB, 1/40 for IP
USP22, ab235923, polyclonal, Abcam, Cambridge, MA, USA, 1/200 for IF
USP33, ab237510, polyclonal, Abcam, Cambridge, MA, USA, 1/1000 for WB
SPI1, ab227835, monoclonal, Abcam, Cambridge, MA, USA, 1/5000 for IHC-P, 1/1000 for WB, 1/30 for IP, 5 μg/25 μg chromatin for CHIP
SPI1, ab88082, monoclonal, Abcam, Cambridge, MA, USA, 1/500 for IF
PD-L1, ab205921, monoclonal, Abcam, Cambridge, MA, USA, 1/1000 for WB, 1/1000 for IHC-P
PD-L1, ab213480, monoclonal, Abcam, Cambridge, MA, USA, 1/500 for IF
Anti-CD8 alpha, ab245118, monoclonal, Abcam, Cambridge, MA, USA, 1/1000 for IHC-P
Anti-CD8 alpha, # GB11068-1, polyclonal, Servicebio, 1/500 for IF
Anti-Granzyme B, ab255598, monoclonal, Abcam, Cambridge, MA, USA, 1/3000 for IHC-P
GAPDH, #5174, D16H11, Cell Signaling Technology, Beverly, MA, 1:1000 for WB
HRP-linked anti-rabbit IgG #7074, Cell Signaling Technology, Beverly, MA, USA, 1:3000 for WB
HRP-linked anti-mouse IgG #7076, Cell Signaling Technology, Beverly, MA, USA, 1:3000 for WB
Normal rabbit IgG, #2729, Cell Signaling Technology, Beverly, MA, USA, 1:100 for IP
Rabbit IgG Isotype Control, BS-0295P, Bioss Antibodies, 5 μg/25 μg chromatin for CHIP
Alexa Fluor 594 goat anti-rabbit IgG, R37117, polyclonal, Life Technologies, Carlsbad, CA, USA, 1:200 for IF
Alexa Fluor 488 goat anti-mouse IgG, R37120, polyclonal, Life Technologies, Carlsbad, CA, USA, 1:200 for IF
Fixable Viability Dye, eFluor™ 780, 65-0865-14, Thermofisher, 1:200 for FCM
Human CD3 Monoclonal Antibody (OKT3), FITC, AB_467057, 14-0037-82, Thermofisher, 1:200 for FCM
Mouse CD3 Monoclonal Antibody (17A2), FITC, AB_467053, 14-0032-82, Thermofisher, 1:200 for FCM
Mouse CD8a Monoclonal Antibody (53-6.7), APC, AB_469335, 17-0081-82, Thermofisher, 1:200 for FCM
Human CD8a Monoclonal Antibody (RPA-T8), APC, AB_10669564, 17-0088-42, Thermofisher, 1:200 for FCM
Human TNF alpha Monoclonal Antibody (MAb11), eFluor™ 450, AB_2043889, 48-7349-42, Thermofisher, 1:200 for FCM
Human Granzyme B Monoclonal Antibody (N4TL33), eFluor™ 450, AB_2724392, 48-8896-42, Thermofisher, 1:200 for FCM
Mouse Granzyme B Monoclonal Antibody (NGZB), eFluor™ 450, AB_11149362, 48-8898-82, Thermofisher, 1:200 for FCM
Human CD279 (PD-1) Monoclonal Antibody (MIH4), PE, AB_10736473, 12-9969-42, Thermofisher, 1:200 for FCM
Mouse CD279 (PD-1) Monoclonal Antibody (J43), PE, AB_466295, 12-9985-82, Thermofisher, 1:200 for FCM

Validation

BLIMP1 (ab198287): Wang WF et al. HSP70-Hrd1 axis precludes the oncorepressor potential of N-terminal misfolded Blimp-1s in lymphoma cells. Nat Commun 8:363 (2017).
BLIMP1 (ab243146): Kallies A et al. Plasma cell ontogeny defined by quantitative changes in blimp-1 expression. J Exp Med 200:967-77 (2004).
BLIMP1 (ab13700): Yuya Okuzaki et al. PRDM14 and BLIMP1 control the development of chicken primordial germ cells. Dev Biol 455:32-41 (2019).
USP22 (ab195289): Wei Y et al. USP22 promotes melanoma and BRAF inhibitor resistance via YAP stabilization. Oncol Lett 21:394 (2021).
USP22 (ab195289): Wang Weichen et al. "Circ-SIRT1 inhibits cardiac hypertrophy via activating SIRT1 to promote autophagy." Cell death & disease 12.11 (2021): 1-13.
USP22 (ab235923)
USP33 (ab237510): Wang Hao et al. Ubiquitin specific peptidase 33 promotes cell proliferation and reduces apoptosis through regulation of the SP1/PI3K/AKT pathway in retinoblastoma." Cell Cycle 20.19 (2021).
SPI1 (ab227835): Liu Z et al. Upregulation of SPI1 during myocardial infarction aggravates cardiac tissue injury and disease progression through activation of the TLR4/NFκB axis. American Journal of Translational Research (2022).
SPI1, ab88082, Zha Z et al. Bu Shen Yi Sui Capsule Alleviates Neuroinflammation and Demyelination by Promoting Microglia toward M2 Polarization, Which Correlates with Changes in miR-124 and miR-155 in Experimental Autoimmune Encephalomyelitis. Oxid Med Cell Longev 2021:5521503 (2021).
PD-L1 (ab205921): Mei J et al. A comparability study of natural and deglycosylated PD-L1 levels in lung cancer: evidence from immunohistochemical analysis. Mol Cancer 20:11 (2021).
PD-L1 (ab213480): Fang W et al. Progranulin induces immune escape in breast cancer via up-regulating PD-L1 expression on tumor-associated macrophages (TAMs) and promoting CD8+ T cell exclusion. J Exp Clin Cancer Res 40:4 (2021).
Anti-CD8 alpha (ab245118): Li S et al. A risk signature with inflammatory and immune cells infiltration predicts survival and efficiency of chemotherapy in gastric cancer. Int Immunopharmacol 96:107589 (2021).
Anti-CD8 alpha (# GB11068-1) Mahmood U et al. A Randomized Phase 2 Study of Pembrolizumab With or Without Radiation in Patients With Recurrent or Metastatic Adenoid Cystic Carcinoma. Int J Radiat Oncol Biol Phys 109:134-144 (2021).
Anti-Granzyme B (ab255598) Scopim-Ribeiro R et al. NSG Mice Facilitate ex vivo Characterization of Ewing Sarcoma Lung Metastasis Using the PuMA Model. Front Oncol 11:645757 (2021).
GAPDH (#5174): Guangping Zhang et. al. NEDD4L inhibits glycolysis and proliferation of cancer cells in oral squamous cell carcinoma by inducing ENO1 ubiquitination and degradation. Cancer Biol Ther (2022).
HRP-linked anti-rabbit IgG (#7074): Krishn SR et. al. Small extracellular vesicle-mediated ITGB6 siRNA delivery downregulates the αVβ6 integrin and inhibits adhesion and migration of recipient prostate cancer cells. Cancer Biol Ther (2022).
HRP-linked anti-mouse IgG (#7076): Guo Y et. al. Eliminating the original cargos of glioblastoma cell-derived small extracellular vesicles for efficient drug delivery to glioblastoma with improved biosafety. Bioact Mater (2022).

Rabbit IgG Isotype Control (BS-0295P) Junjie Zhou et al. TNIP3 is a novel activator of Hippo-YAP signaling protecting against hepatic ischemia/reperfusion injury. Hepatology. 2021 10;74 (4), 2133-2153.
Alexa Fluor 594 goat anti-rabbit IgG (R37117): Belinda S Hall et. al. Inhibition of the SEC61 translocon by mycolactone induces a protective autophagic response controlled by EIF2S1-dependent translation that does not require ULK1 activity. Autophagy (2022).
Alexa Fluor 488 goat anti-mouse IgG (R37120): Wen-Ling Mou et. al. LPS-TLR4/MD-2-TNF-α signaling mediates alcohol-induced liver fibrosis in rats. J Toxicol Pathol (2022).
CD3 Monoclonal Antibody (OKT3): Baeuerle PA et.al. Synthetic TRuC receptors engaging the complete T cell receptor for potent anti-tumor response. Nature communications (2019).
CD3 Monoclonal Antibody (17A2): Baharak Bahmani et.al. Targeted delivery of immune therapeutics to lymph nodes prolongs cardiac allograft survival. The Journal of clinical investigation (2018).
CD8a Monoclonal Antibody (53-6.7): John E Pearl et.al. Nitric oxide inhibits the accumulation of CD4+CD44hiTbet+CD69lo T cells in mycobacterial infection. European journal of immunology (2012).
CD8a Monoclonal Antibody (RPA-T8): Jinyun Chen et.al. Umbilical Cord-Derived Mesenchymal Stem Cells Suppress Autophagy of T Cells in Patients with Systemic Lupus Erythematosus via Transfer of Mitochondria. Stem cells international (2020).

TNF alpha Monoclonal Antibody (MAb11): Yael Kusne et.al. Targeting aPKC disables oncogenic signaling by both the EGFR and the proinflammatory cytokine TNFα in glioblastoma. Science signaling (2014).

Granzyme B Monoclonal Antibody (NGZB): Teck-Hui Teo et.al. Caribbean and La Réunion Chikungunya Virus Isolates Differ in Their Capacity To Induce Proinflammatory Th1 and NK Cell Responses and Acute Joint Pathology. Journal of virology (2015).

CD279 (PD-1) Monoclonal Antibody (MIH4): Qian Niu et.al. Enhanced IL-6/phosphorylated STAT3 signaling is related to the imbalance of circulating T follicular helper/T follicular regulatory cells in patients with rheumatoid arthritis. Arthritis research & therapy (2018).

CD279 (PD-1) Monoclonal Antibody (J43): Ansari MJ et.al. The programmed death-1 (PD-1) pathway regulates autoimmune diabetes in nonobese diabetic (NOD) mice. The Journal of experimental medicine (2003).

All antibodies involved in the current study are commonly available from commercial vendors. All antibody clones have been validated by the vendors. Validation data are available on the manufacturer's website.

Eukaryotic cell lines

Policy information about cell lines and Sex and Gender in Research

Cell line source(s)	Hep3B (HB-8064), Hepa1-6 (CRL-1830), and 293T (CRL-3216) cell lines were purchased from the American Type Culture Collection (ATCC). Huh7 cell line (JCRB0403) was purchased from Japanese Cancer Research Resources Bank (JCRB). H22 cell line (GDC0091) was purchased from China Center for Type Culture Collection (CCTCC).
Authentication	The cell lines were authenticated by short tandem repeat (STR) profiling.
Mycoplasma contamination	All cell lines used were mycoplasma free.
Commonly misidentified lines (See ICLAC register)	None as far as we know.

Animals and other research organisms

Policy information about studies involving animals; ARRIVE guidelines recommended for reporting animal research, and Sex and Gender in Research

Laboratory animals	4-week-old BALB/c nude male mice and C57BL/6 mice were employed to perform in vivo experiment. Mice were housed in specific pathogen free (SPF) conditions, dark/light cycles: 12-hours light/12-hour dark (150-300 lux), ambient temperature 20-26℃ and humidity 40%-70%, ventilated four times per hour.
Wild animals	We did not use any wild animals.
Reporting on sex	Sex and gender were not considered in our study design.
Field-collected samples	Studies did not include samples collected from the field
Ethics oversight	All the animal studies were approved by the Institutional Animal Care and Use Committee of Nanjing Medical University and conducted according to protocols approved by the Ethical Committee of Nanjing Medical university.

Note that full information on the approval of the study protocol must also be provided in the manuscript.

Flow Cytometry

Plots

Confirm that:

- [x] The axis labels state the marker and fluorochrome used (e.g. CD4-FITC).
- [x] The axis scales are clearly visible. Include numbers along axes only for bottom left plot of group (a 'group' is an analysis of identical markers).
- [x] All plots are contour plots with outliers or pseudocolor plots.
- [x] A numerical value for number of cells or percentage (with statistics) is provided.

Methodology

Sample preparation	Freshly resected mouse tumors or tumors from patients with HCC of comparable size (~ 100 mm3) were dispersed into single-cell suspensions. The cells were incubated with the appropriate antibodies at room temperature for 30 min. After washing twice in PBS, the samples were analyzed using flow cytometry.
Instrument	Flow cytometry (Beckman Coulter, USA).
Software	FlowJo v10.7 (Tree Star, California, USA).
Cell population abundance	At least 30000 cells in one cube and 10000 cells were analyzed per sample.
Gating strategy	Gating was performed based on identifying a distinct population in FSC and SSC plots.

- [x] Tick this box to confirm that a figure exemplifying the gating strategy is provided in the Supplementary Information.
